# Activity in the Arbuscular Mycorrhizal Hyphosphere Warning Neighbouring Plants

**DOI:** 10.3389/fpls.2019.00511

**Published:** 2019-04-18

**Authors:** Carmina Cabral, Bernd Wollenweber, Carla António, Sabine Ravnskov

**Affiliations:** ^1^Aarhus University, Department of Agroecology, Slagelse, Denmark; ^2^Plant Metabolomics Laboratory, Instituto de Tecnologia Química e Biológica António Xavier-Universidade NOVA de Lisboa (ITQB NOVA), Oeiras, Portugal

**Keywords:** arbuscular mycorrhizal fungal networks, gene-expression, hyphosphere, mycorrhiza-associated bacteria, PR proteins, primary metabolism, SAR

## Abstract

Pathogen infections of the phyllosphere have been investigated in detail, however, the changes induced by these infections on the arbuscular mycorrhizal hyphosphere, and the consequent signalling to the neighbouring plants have been scarcely investigated. Here, our objectives were to document that *B.fabae* infection of connected *Vicia faba* plants resulted in changes in the metabolism and microbial community of the hyphosphere, confirming the induction of plant defence in connected plants through gene-expression evaluations. Infected plants were challenged with *B. fabae* for 72 h. Changes in gene-expression of pathogenesis-related proteins 1,2, and 5 (*PR1*, *PR2*, *PR5*) of both infected- and non-infected plants were analysed, to confirm signalling through the hyphosphere. The primary metabolic profiles and changes in the level of microbiota in the hyphosphere were assessed. Changes in expression of *PR1*, *PR2*, and *PR5* genes occurred in the neighbouring plants 24 hours after infection. Mannitol levels decreased in presence of AMF. A decrease in the level of actinobacteria in the hyphosphere of infected plants was detected. We conclude that *B.fabae* infection induced a signalling event through the AM hyphosphere, confirmed by changes in defence gene-expression in non-infected neighbouring plants, influenced primary metabolic activity of-, and affected the microbial composition within-, the AM hyphosphere.

## Introduction

Arbuscular mycorrhizal fungi (AMF) are mutualistic biotrophic fungi, which form symbiotic associations with around 72% of higher plants (Brundrett and Tedersoo, 2018), depending on the plants for carbohydrate supply and providing mineral nutrients (Smith and Smith, 2011). Establishment of the AM symbiosis has been reported to influence the nutritional status of the plant (Chen and Zhao, 2009; Bati et al., 2015; Ravnskov and Larsen, 2016) and to improve disease tolerance (Whipps, 2004). In the AM symbiosis, hexoses from the host plant are transferred to the intraradical mycelium and converted into storage lipids, which are then translocated into the extraradical mycelium (Bago et al., 2002, 2003). The fungus, being exclusively dependent on carbon acquired from the host plant, cannot absorb exogenous hexoses (Bago et al., 2003). Moreover these fungi are also dependent on the plant for fatty-acid supply (Bravo et al., 2017). Thus, it may be plausible that changes in host plant metabolism are reflected in changes in primary metabolism of the AMF symbiont (Ravnskov et al., 2003; Cabral et al., 2018).

The low host specificity and ability to anastomose across AMF species results in the formation of an intricate AM mycelial network that connects plants underground (Giovannetti et al., 2004; Mikkelsen et al., 2008). These underground mycelial networks have been studied with regards to nutrient and water exchanges between plants (Weremijewicz et al., 2016). Their role as ‘communication channels’ has also been the object of interest. Several studies have proposed that a (to date unspecified) signal travels between interconnected plants, after a biotic stressor is applied to one of the plants (Song et al., 2010, 2014; Babikova et al., 2013; Cabral et al., 2018). These studies have established that the timing and type of plant defence response induction varies, dependent of the nature of the stressor (pest or pathogen) (Johnson and Gilbert, 2015; Cabral et al., 2018). Using *Aphis fabae* as a stressor on *Vicia faba* plants, we have also shown that as a result of the signalling process between challenged and neighbouring plants, the primary metabolic profile of the hyphosphere itself was modified (Cabral et al., 2018).

As sessile organisms, plants possess a multi-layered immune system that employs both constitutive and inducible defence strategies when faced with an antagonist (Jones and Dangl, 2006; Gruner et al., 2013). These layers can be classified, in accordance to the zig-zag model, as a response against pathogen-associated molecular patterns (PAMPS), namely pattern triggered immunity (PTI), and an effector-triggered response (ETI) (Jones and Dangl, 2006). As the first order of defence, PTI consists of several physical modifications, (i.e., callose deposition) and biochemical responses, such as Ca^2+^ signalling and the induction of salicylic acid (SA) or jasmonic acid (JA) dependent defence pathways (Garcia-Brugger et al., 2006). In order to deploy these defence strategies, plants have to shift resources (metabolites, energy and reduction equivalents) from general metabolism towards defence, ultimately affecting plant growth and development (Berger et al., 2007; Bolton, 2009).

In response to a pathogen attack, the plant synthesises several pathogenesis-related proteins (PRs), which accumulate both locally and systemically (Aoun, 2017). These proteins have different functions, and their encoding genes are part of different transduction pathways (Kunkel and Brooks, 2002), i.e., *PR1*, *PR2*, and *PR5* are induced by SA, while *PR3*, *PR4*, and *PR12* are induced by JA. Furthermore, several of the PR proteins have been shown to have direct antimicrobial and antifungal properties (Mauch et al., 1988). These have been proposed to be a part of a systemic acquired resistance (SAR) response occurring after localised microbial infection, where either PTI or ETI are triggered, and SAR markers, such as SA, *PR1*, *PR2* and *PR5*, are expressed (Gruner et al., 2013). At the phyllosphere level, SAR acts in a way of priming the surrounding leaves to a local infection. Mechanistically, it shares many characteristics with mycorrhiza-induced resistance (MIR), where AMF have been reported to suppress pests and plant diseases through induction of systemic resistance (Cameron et al., 2013). MIR has been reported to be akin to a SAR-like priming of the SA and JA dependent plant defence pathways, conferring protection against a wide range of antagonists (Vannette and Hunter, 2009; Cameron et al., 2013). Furthermore, as AMF is known to influence biological activity in the rhizosphere (Azcón-Aguilar and Barea, 1997; Barea et al., 2002) and in its own hyphosphere (Albertsen et al., 2006; Welc et al., 2010; Cabral et al., 2018), the role of the mycorrhiza-associated bacteria in SAR cannot be discarded (Frey-Klett et al., 2007).

The microbes associated with the AM mycelium, constituting the hyphosphere, are instrumental for the plant beneficial traits of AMF (Li et al., 2007). The AM external mycelium has been reported to have both a suppressive effect on its surrounding microbiota (Welc et al., 2010), as well as to increase bacterial presence (Albertsen et al., 2006). This increase was reported for different genera, such as Gram-negative- (Battini et al., 2016), and Gram-positive bacteria (Battini et al., 2016; Lasudee et al., 2018). Furthermore, several strains of actinobacteria have been reported to be associated with AMF spores (Lasudee et al., 2018), and their activity as plant growth promoters and biocontrol agents has been investigated (Li et al., 2007; Battini et al., 2016). Therefore, the functional compatibility, not only between AMF genotype and host plant species, but also, its influence on the surrounding microbiota should be further investigated.

Here, we hypothesised that *B. fabae* infection of the leaves of *Vicia faba* would initiate a signalling event between infected- and non-infected neighbouring plants connected by the AM hyphosphere. As plant defence mechanisms against pest and pathogens differ, it was further hypothesised that changes in the metabolic activity of the hyphosphere connecting the plants would differ from previously detected changes after aphid infestation of the phyllosphere. In addition, changes in the metabolic activity in the hyphosphere were expected to influence the composition of its microbial community. Therefore, the objectives of the current study were: (i) to ascertain that a *B. fabae* infection of the leaves of infected plants elicits a response in the leaves of non-infected neighbouring plants; (ii) to characterise the primary metabolic profiles of the AM hyphosphere connecting plants after *B. fabae* infection; and iii) to compare changes to the microbial communities in the AMF hyphosphere of *B. fabae* affected- and unaffected plants.

## Materials and Methods

### Experimental Setup and Growth Conditions

**FIGURE 1:**
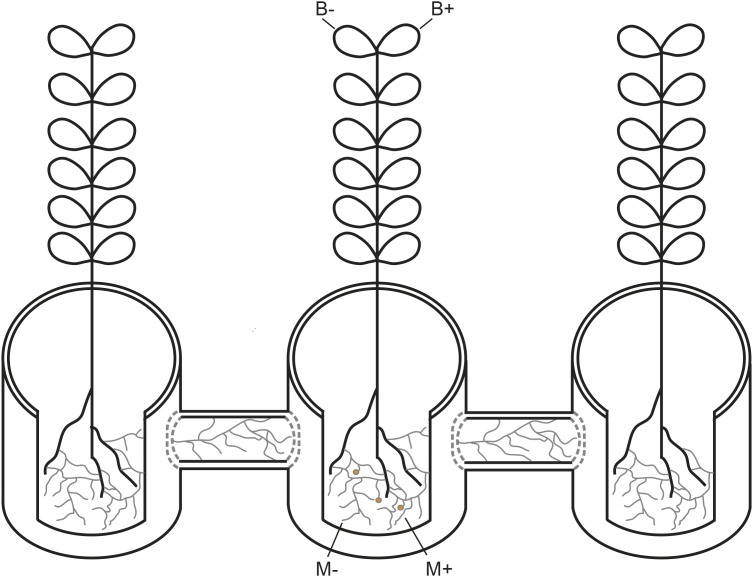
Schematic representation of the plant growth system used in the current study. AM networks via the challenged plant were (M+) established or not (M−) in neighbouring plants. Challenged plants were infected with *Botrytis fabae* (B+) or not (B−).

A glasshouse experiment was performed at Aarhus University, Denmark (55°19°N, 11°24°E) from October to December 2017. The experiment was based on a full factorial design, assessing two factors and their respective combinations: inoculation of plants with AMF (M+) or not (M−), and infection of plants with *Botrytis fabae* Sard. (B+) or not (B−), where each factor combination comprised five replicates, the AMF network was either established (M+) or not (M−) through the hyphal compartments towards the neighbouring plants (Figure 1) The faba bean (*Vicia faba* L.) cultivar “Boxer” was used due to its uniform growth. A total of 20 growth systems were set up as described in detail in (Cabral et al., 2018), where a 3.4 L plant pot is placed at the centre and connected to two 3.4 L plant pots on either side, by 1.2 L hyphosphere compartments. Plant pots were filled with a 1:2 soil:sand mixture (one part of low P soil for 2 parts of sand), twice disinfected at 80°C for 24 hours, while a 1:3 proportion of soil:sand mixture was used for the hyphosphere compartment. The hyphosphere compartments were filled with 800 g of 1:3 disinfected soil:sand mixture, creating buffer zones at the end of each hyphosphere compartment connecting challenged and neighbouring plants. These buffer zones consisted of approximately 1,5 cm in each end, and were discarded at sampling, to account for root exudation. After filling, the end of each hyphosphere compartment was closed with a 20 μm nylon mesh. The 1:2 and 1:3 soil: sand disinfected growth substrate were fertilised with macro and micronutrient solutions, excluding P, as according to protocol described in (Cabral et al., 2016),posteriorly watered to 100% soil relative water content, and left for a week to allow for inoculum incubation. Seeds were sown after scarification, sterilisation and incubation at 24°C during two days, which allowed for confirmation of radicle emergence. Plants were watered every day to maintain soil at 60% soil relative water content. Growth conditions were set at 16:8 light: dark period and a day: night temperature of 20:18°C.

### *Botrytis fabae* Propagation and Spore Production

A mix of six different isolates were used for *B. fabae* inoculation. The fungi were isolated from different faba bean fields around Denmark and the identity of the isolates was verified by sequencing the HSP60 and G3PDH regions (Primers against Genbank. AJ716074 and AJ705013, respectively) (Supplementary Table 2) (Staats et al., 2005). *B. fabae* was maintained in culture by replating on agar. The following media recipe was used: 500 ml of an extract made by cooking 125 g *Vicia faba* L. leaves, 7.5 g sucrose, 15 g NaCl and 7.5 g of agar. A total of 30 agar plates grown with *B. fabae* for ten days was used to prepare a 500 ml spore suspension (3.5 × 10^4^ spores per ml) in autoclaved H_2_O added with 0.5 ml of Tween 20. After *B. fabae* inoculation, fifty autoclaved clip-cages were dipped in this spore suspension for 30 s and placed on agar plates, whilst the 50 control clip-cages were dipped in autoclaved water and also placed on agar plates prepared as described above. All clip-cages on agar were incubated in the dark for 48 h at 22°C, before being moved to 22°C in a 12:12 h light: dark UV light room, where these were kept for seven days, to assure successful spore production.

### *Botrytis fabae* Sard. Challenge

Plants were challenged with *B. fabae* during 72 h eight weeks after sowing, when the plants reached physiological stage 39 (Meier et al., 2009). For the challenge, clip-cages containing *B.fabae* mycelia and spores were placed on the 3rd, 4th, 6th, 7th, and 8th leaves of the (B+) plants, while empty clip-cages were used for the (B−) plants on the same leave numbers. After pathogen inoculation the challenged plants were immediately sealed with airtight polyethylene bags to avoid airborne communication between plants as described in (Cabral et al., 2018). During the challenge, leaves were collected from one of the two connected neighbouring plants and from extra challenged plants (separated from the main experiment, grown under the same conditions) at 24, 48, 60, and 72 hours post infection (hpi). Leaves were shock-frozen in liquid N, freeze-dried and ground for posterior analyses. At harvest time, the leaves with clip-cages were shock-frozen, freeze-dried and ground, for *B. fabae* quantification through qPCR analysis.

### Harvest, Sampling and Dry Weights

When plants reached physiological stage 39 (Meier et al., 2009) at week 9, B+ plants were inoculated with *B. fabae* and were harvested after 72 h following the steps described in (Cabral et al., 2018) : dislodging of the hyphosphere compartments, followed by freeze-shocking and harvest of the above and belowground plant parts. All samples, except samples for AMF root colonisation analysis, were frozen, lyophilised and kept at −20°C for further analysis.

### Root Staining, Root Length and AMF Colonisation Analysis

After harvest of aboveground plants, all root material was carefully rinsed with tap water and cut into 1 cm pieces. Samples of 0.8 g fresh weight were taken for determination of AMF colonisation. These samples were cleared in 10 % KOH and stained in 5 % inkblue acetic acid, adapted from (Vierheilig et al., 1998) and stored in glycerol. AM fungal colonisation was assessed by microscopy at 16x magnification, using the point-intersect method (McGonigle et al., 1990), and AM fungal structures were classified as: intraradical hyphae, arbuscules and vesicles. The remaining root material was frozen, freeze-dried, ground and stored at −20°C, prior to further analysis.

### Quantification of *B. fabae* Infection on Infected Plant Leaves

*Botrytis fabae* infection was quantified by qPCR (Supplementary Table 1). DNA was extracted from infected plant leaves using a NucleoMag Plant extraction kit (Macherey-Nagel 744400.4) and following manufacturer’s instructions. Primers against the *B. fabae* 5.8S rRNA gene Genbank accession n.KX074007, one of the conserved regions of *B.fabae*, were generated using the software Primer3 (Rozen and Skaletsky, 2000) (Supplementary Table 2). The qPCR reactions were carried out using 1 μl (10 mM) the primer pair, 2 μl DNA template, 6 μl of the SYBR green master mix (Quanti Tech SYBR Green kit, Qiagen, GmbH Hilden, Germany) diluted to a final volume of 12 μl with RNase-free water. In the negative control, DNA template was replaced by RNase free water. The reactions were performed on an Applied Biosystems^®^ ViiA^TM^ 7 Real-Time PCR System. The programme used for qPCR was as follows: heating step of 2 min at 50°C, 10 min initial denaturation at 95°C, followed by 40 cycles of denaturation for 15 s at 95°C, annealing for 1 min at 60°C. The fluorescence signal was measured immediately after incubation for 1 min at 60°C following the annealing step. At the end of the cycles, melting temperatures of the PCR products were determined between 60°C and 95°C. A standard curve with known quantities of *B.fabae* DNA was established from the cultured fungi (Supplementary Figure 1), to allow quantification of infection on plant leaves. The analysis was carried out for 5 independent biological replicates and 2 technical replicates per reaction.

### Gene-Expression Assays in Faba Bean Plants

Changes in expression of the genes: *PATHOGENESIS-RELATED PROTEIN 1* (*PR1*), *PATHOGENESIS-RELATED PROTEIN 2* (*PR2*) and *PATHOGENESIS-RELATED PROTEIN* 5 (*PR5*) were evaluated by two step RT-qPCR, after RNA extraction from both neighbouring and challenged plant leaves in each growth system. These were compared to the reference gene *ELONGATION-FACTOR 1-A* (*ELF1-α*), for normalisation of expression, using the 2^−ΔCT^ method (Livak and Schmittgen, 2001). Total RNA was extracted from faba bean leaves, using an RNA NucleoMag Kit (744350.1 MACHEREY-NAGEL GmbH & Co. KG), according to manufacturer’s instructions. First strand cDNA was synthesised from 1 mg of total RNA using a High activity cDNA Reverse Transcription Kit (4368814 Applied Biosystems^®^) according to the manufacturer’s instructions, RT- controls were added to account for gDNA contamination. The primers for target genes *PR1*, *PR2*, *PR5* and *Elf1*-α, were selected from the literature (Gutierrez et al., 2011; El-Komy, 2014) and designed with the software Primer3 (Rozen and Skaletsky, 2000) (Supplementary Table 2). The RT-qPCR reactions were carried out as described above for *B.fabae* quantification, no amplification controls (NAC) were added for each reaction. No less than three biological replicates (from different plants) were independently carried out by treatment and time-point, and two technical replicates (2 reactions with template from same plant) of each were considered for measurements of relative gene expression.

### Extraction and Relative Quantification of Primary Metabolites From Root-Free Compartments

Primary metabolites were extracted using a methanol/chloroform extraction protocol adapted from (Lisec et al., 2006). A total of 20 g dry weight of finely homogenised hyphosphere substrate was weighed into 50 ml falcon tube and diluted in 12000 μl ice-cold 100% (v/v) methanol with ribitol (0.2 mg ml^−1^ ribitol in water) as an internal standard. The mixture was vortex-mixed, incubated for 15 min on an orbital shaker (Brunswick^TM^ Innova^®^ 44) at 250 rpm for 15 min at 70°C. Subsequently, each tube was centrifuged at room temperature at 14,000 *g* for 10 min. The supernatant was transferred to a new 15 ml falcon tube, mixed with 1850 μl chloroform and 3750 μl H_2_O, and vortex mixed. Samples were centrifuged at 4000 rpm for 15 min at room temperature. A total of 10000 μl of the polar (upper) aqueous/methanol phase were evaporated to dryness using a centrifugal concentrator (Vacufuge Plus, Eppendorf, Hamburg, Germany) and stored at −80°C. Primary metabolites were derivatized and analysed using an established GC-TOF-MS protocol (Lisec et al., 2006) at the Biochemistry and Plant Biotechnology Laboratory (University of Malaga, Spain). The obtained GC-TOF-MS files (cdf format) were subsequently evaluated using AMDIS (Automated Mass Spectral Deconvolution and Identification System) software (v 2.71). Primary metabolites were annotated using the TagFinder software (Luedemann et al., 2008) and a reference library of ambient mass spectra and retention indices from the Golm Metabolome Database^1^ (Supplementary Table 3) (Kopka et al., 2005; Schauer et al., 2005). GC-TOF-MS relative primary metabolite levels were normalised to the internal standard (ribitol) and calculated according to specific dry weight of the samples.

### Whole-Cell Fatty-Acid Signature Analysis of Microbes in the AM Hyphosphere

One gram of sample from each root-free compartment connecting challenged and neighbouring plants was used for whole-cell fatty acid extraction. The samples were saponified with a NaOH-methanol mixture, methylated with HCl-methanol, and finally extracted with hexane/methyl tert-butyl ether (MTBE). The MTBE was amended with 33.75 μg of nanodecanoic acid methyl esther (19:0) as an internal standard. The extract was then washed with NaOH, evaporated under N2 stream, resuspended in 100 μL of hexane and injected into an Agilent 6890 plus GC. Oven temperature increased following a 5°C/min ramp, from 170 to 260°C, afterwards following a 40°C/min ramp until it reached 310°C. A phenyl-siloxane (2.5%) column was used (25 m long, 200 lm ID, 0.33 lm film), hydrogen and nitrogen were used as carrier and make-up gases. A flame ionisation detector was used, fed by a hydrogen–air mixture. Fatty acids were analysed through the MIDI microbial identification protocol (Sherlock version 4.5 MIDI, Microbial ID, Newark, DE, United States).

### Statistical Analysis

Data analyses were performed using R-software (R Development Core Team, 2017). Values expressed in percentage, relative values as well as ratios were log-transformed, before statistical analysis, in order to fit the normality and homoscedasticity assumptions of the analysis of variance (ANOVA). Homoscedasticity and normality were tested using Bartlett’s and Shapiro-Wilks tests, respectively. Linear models followed by two-way ANOVA were used to assess differences between factors, and linear mixed models, followed by type III ANOVA with Satterthwaite’s method were used to account for random effects in repeated measures. *Post-hoc* analysis for mean comparison at 95% confidence interval was performed in terms of Tukey’s HSD_0.05_ and Benjamini-Hochberg false discovery rate (fdr) correction on the *P*-values (Benjamini and Hochberg, 1995), to assess differences between treatments. The R software packages: “corrplot”(Wei and Simko, 2017), “FactoMineR”(Le et al., 2008), “missMDA” (Josse and Husson, 2016), “factoextra” (Kassambara and Mundt, 2017) and “ggplot2” (Wickham, 2010) were used for data analysis and to perform the Principle component analysis on the primary metabolite levels. Figures were made using SigmaPlot Version 11.0 (Systat Software, San Jose, CA, United States).

## Results

### Plant Biomass and AMF Colonisation

No differences in shoot dry weight (M−:10.07 ± 0.52, M+:9.71 ± 0.49), shoot length (M−:0.98 ± 0.04,M+:0.86 ± 0.04) or root dry weight (M−:0.95 ± 0.05,M+:0.912 ± 0.02) were detected between M− and M+ plants in challenged plants (Supplementary Table 1). Likewise, in neighbouring plants, no differences between M− and M+ plants were detected in: shoot dry weight (M−:9.38 ± 0.34, M+:9.96 ± 0.44), shoot length (M−:0.88 ± 0.03,M+:0.90 ± 0.02) (Supplementary Table 1). No AMF structures were detected in M− plants. M+ challenged plants exhibited a higher percentage total AMF colonisation, intraradical hyphae, arbuscules and vesicles than neighbouring plants (Table 1). There was no significant effect of *B.fabae* infection on the abovementioned traits.

**Table 1:** Percentages of total colonisation and fungal structures of arbuscular mycorrhizal fungi (AMF) in *Vicia faba* root systems after harvest.

M+			
**Percentage of AMF structures (%)**	**Challenged**	**Neighbouring**	**Plant Type**
Total colonisation	0.89 ± 0.01a^1^	0.53 ± +0.03b	^∗∗∗^
Intraradical hyphae	0.44 ± 0.02a	0.37 ± 0.01b	^∗^
Arbuscules	0.34 ± 0.01a	0.15 ± 0.02b	^∗∗∗^
Vesicles	0.12 ± 0.01a	0.01 ± 0.00b	^∗∗∗^

*Challenged plant columns represent plants from the central compartment inoculated (M+) with AMF, where both plants infected with Botrytis fabae (B+) or not (B−) are grouped. Neighbouring plant columns represent plants from the neighbouring compartments, in which AM networks via the donor plant were (M+) established. Values are means ± standard error (SE) (Challenged plants: N = 10; Neighbouring plants: N = 17). Statistical significance between treatments indicated by ^∗^ (P < 0.05), ^∗∗^ (P < 0.01), ^∗∗∗^ (P < 0.001), ns (not significant). ^1^In each row, values followed by the same letter are not significantly different by Tukey’s HSD_0.05_ (honestly significant difference) with a P-value BH-fdr (Benjamini-Hochberg false discovery rate) correction.*

### *Botrytis fabae* Infection on Inoculated Leaves

Both M− and M+ infected plants were grouped for the determination of *B.fabae* infection in inoculated leaves (Supplementary Figure 1). In B+ plants, 0.0019 ng/g^−1^ sample of *B. fabae* were detected inchallenged leaves inoculated with *B. fabae* (B+), 72 hpi, while no infection was detected in B− plants.

### Changes in Gene Expression of PR1, PR2 and PR5 in Faba Bean Challenged and Neighbouring Leaves

**FIGURE 2:**
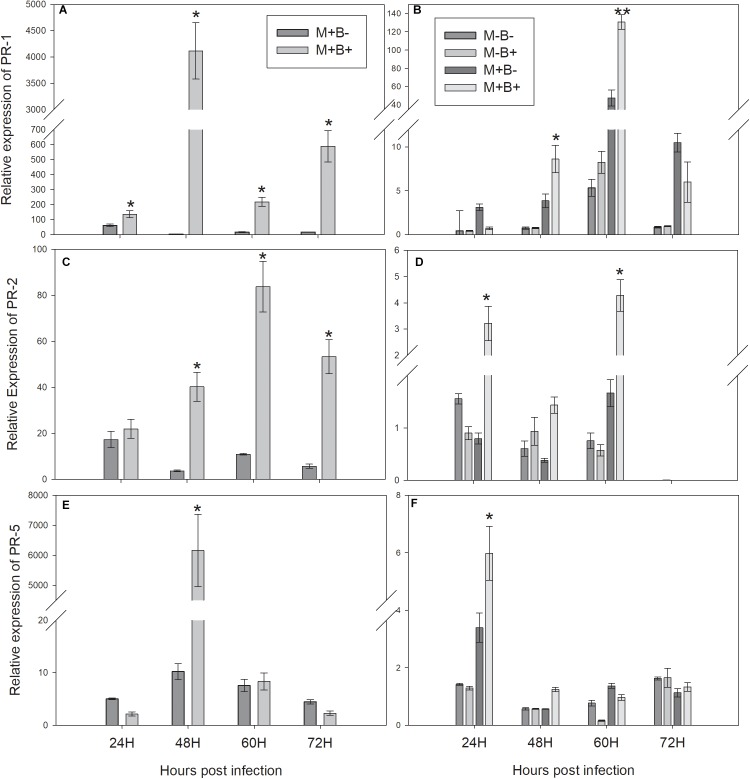
Relative-expression of the measured PR protein coding genes at 24, 48, 60 and 72 hours post *Botrytis fabae* infection. **(A)** Relative expression of *PATHOGENESIS-RELATED PROTEIN 1 (PR1)* in challenged plants. **(B)** Relative expression of *PR1* in neighbouring plants **(C)** Relative expression of *PATHOGENESIS-RELATED PROTEIN 2 (PR2)* in challenged plants. **(D)** Relative expression of *PR2* in neighbouring plants. **(E)**: Relative expression of *PATHOGENESIS-RELATED PROTEIN 5 (PR5)* in challenged plants. **(F)**: Relative expression of *PR5* in neighbouring plants. AM networks via the donor plant were (M+) established or not (M−) in neighbouring plants. Challenged plants were infected with *B.fabae* (B+) or not (B−). Statistical significance between treatments inside timepoints indicated by ^∗^*P* < 0.05, ^∗∗^*P <* 0.01, ^∗∗∗^*P* < 0.001, ns (not significant), *N* = 5. Error bars represent +− SE of the mean.

The expression of *PR1* increased in B+ directly infected plants at 24 hpi, peaking at 48 hpi and remaining higher than in B− plants throughout 60 and 72 hpi (Figure 2A). Consequently, *PR1* expression increased in M+B+ neighbouring plants at 48 hpi, peaking at 60 hpi and decreasing at 72 hpi (Figure 2B). *PR2* expression in B+ directly infected plants increased at 48 hpi, peaking at 60 hpi and remaining significantly higher at 72 hpi (Figure 2C) In the M+B+ neighbouring plants, *PR2* expression increased at 24 hpi, decreasing at 48 hpi and peaking at 60 hpi (Figure 2D). *PR5* expression increased in directly infected plants at 48 hpi (Figure 2E). In M+B+ neighbouring plants, *PR5* expression increased at 24 hpi, decreasing at 48 hpi (Figure 2F).

### Primary Metabolic Profiles in Root-Free Compartments

In total, nine putative primary metabolite signatures were found to be present in the root-free compartments: methionine, glucose, mannose, rhamnose, xylose, trehalose, mannitol, benzoic acid and urea (Table 2), punctual differences in specific metabolites were found to be significant (Table 2). Mannitol levels decreased significantly with AMF colonisation and *B. fabae* infection. Urea levels showed a trend towards increasing with *B. fabae* infection (Table 2), seemingly decreasing in the M+B+ root-free compartments (Table 3).

**Table 2:** Fold changes of relative primary metabolite levels relative to M−B− treatment, normalised to the internal standard (ribitol) and dry weight of the samples, in hyphosphere compartments.

Classes	Metabolites	M−B−	M−B+	M+B−	M+B+	AMF	*B. fabae*	AMF^∗^ Botrytis
Amino acids	Methionine	1.00 0.18a^1^	1.64 0.42a	1.68 0.29a	1.05 0.53a	ns	ns	0.06.
Sugars and Sugar	Glucose	1.00 0.09a	2.48 1.62a	3.11 2.05a	1.03 0.60a	ns	ns	ns
alcohols	Mannose	1.00 0.15a	1.05 0.26a	1.17 0.22a	1.10 0.63a	ns	ns	ns
	Rhamnose	1.00 0.12a	0.82 0.10a	0.96 0.14a	0.92 0.46a	ns	ns	ns
	Xylose	1.00 0.13a	0.89 0.18a	1.07 0.22a	0.95 0.48a	ns	ns	ns
	Trehalose	1.00 0.09a	1.35 0.28a	1.36 0.44a	0.82 0.47a	ns	ns	ns
	Mannitol	1.00 0.27a	0.35 0.17b	0.20 0.02b	0.19 0.11b	^∗^	^∗^	0.06.
Others	Benzoic acid	1.00 0.30a	0.91 0.27a	0.74 0.10a	0.94 0.54a	ns	ns	ns
	Urea	1.00 0.14a	2.45 0.57a	1.91 0.29a	1.72 1.0a	ns	0.06.	^∗^

*Arbuscular mycorrhizal fungal (AMF) networks via the challenged plant were (M+) established in neighbouring plants or not (M−). Challenged plants in these systems were (B+) infected with Botrytis fabae or not (B−). Values are means ± standard error (SE), N = 5. Statistical significance between treatments indicated by ^∗^ (P < 0.05), ^∗∗^ (P < 0.01), ^∗∗∗^ (P < 0.001), trends are indicated by:. (0.05 > P ≤ 0.07), ns (not significant). ^1^In each row, values followed by the same letter are not significantly different by Tukey’s HSD_0.05_ (honestly significant difference) with a P-value BH-fdr (Benjamini-Hochberg false discovery rate) correction.*

**Table 3:** Microbial fatty-acid signature analysis of hyphosphere compartments connecting *Vicia faba* L. plants.

Signature fatty-acids	M−B−	M−B+	M+B−	M+B+	AMF	Botrytis	AMF^∗^Botrytis
Arbuscular mycorrhizal fungi	0.024 0.002b	0.021 0.002b	0.029 0.002a	0.027 0.02a	^∗∗^	ns	ns
Saprotrophic fungi	0.013 0.003a	0.018 0.009a	0.011 0.001a	0.001 0.007a	ns	ns	ns
Gram-negative bacteria	0.022 0.00a	0.026 0.003a	0.024 0.005a	0.025 0.01a	ns	ns	ns
Gram-positive bacteria	0.081 0.008a	0.078 0.006a	0.077 0.006a	0.074 0.005a	ns	ns	ns
Actinobacteria	0.013 0.002ab	0.014 0.003ab	0.017 0.004a	0.008 0.001b	ns	^∗^	0.07.

*Arbuscular mycorrhizal fungal (AMF) networks via the challenged plant were (M+) established in neighbouring plants or not (M−). Challenged plants in these systems were (B+) infected with Botrytis fabae or not (B−). Values are means ± standard error (SE). N = 5. Statistical significance between treatments indicated by ^∗^(P < 0.05). ^∗∗^(P < 0.01). ^∗∗∗^(P < 0.001). trends are indicated by:. (0.05 > P ≤ 0.07). ns (not significant). ^1^In each row. values followed by the same letter are not significantly different by Tukey’s HSD_0.05_ (honestly significant difference) with a P-value BH-fdr (Benjamini-Hochberg false discovery rate) correction.*

### Fatty-Acid Signature Analysis

Whole-cell fatty acid signature analysis allowed identification of Gram-positive bacteria (15:0iso, 15:0anteiso, 16:0iso, 17:0iso and 17:0anteiso), Gram-negative bacteria (12:0 2OH, 12:0 3OH, 16:0 2OH, 16:0 3OH, cy17:0 and cy19:0), as well as saprotrophic fungi (18:2ω6,9), actinobacteria (Me16:0, Me17:0, Me18:0) and AMF (16:1ω5) (Table 3). There was no effect of *B. fabae* infection in regards to the relative levels of AMF in hyphosphere compartments (Table 3). There was an increase in the relative levels of AMF, relative to AMF colonisation in the hyphosphere compartments (Table 3). The relative levels of Actinobacteria decreased in M+B+ root-free compartments, in comparison with M+B− (Table 3). Infection of challenged plants with *B. fabae* did not influence the relative levels of Gram-negative bacteria, Gram-positive bacteria or fungi in the hyphosphere (Table 3).

## Discussion

We have shown that the induction of defence in the interconnected *B. fabae* infected plants influenced the primary metabolic activity of the connecting AMF hyphosphere, which in turn influenced the composition of its microbiota. The induction of defence in the interconnected plants was consequential, indicating that there was a (to date unknown) signalling event travelling through the hyphosphere, in accordance to previous studies (Song et al., 2010; Babikova et al., 2013; Cabral et al., 2018). The upregulation of the measured plant-defence genes in neighbouring plants was faster than in previous studies, where different pathogens and pests were used as stress inducers (Song et al., 2010, 2014; Babikova et al., 2013; Cabral et al., 2018). This faster induction of the defence response in the neighbouring plants might be due to the faster induction of the SA-dependent PR-protein genes (*PR1*, *PR2*, and *PR5*) (Aoun, 2017) in the infected plants, shifting the timing of the perception of the signalling by the interconnected neighbouring plants. These shifts in the timing of signalling perception might be due to the different host genotype, AMF species and pest/pathogen combinations in the different studies as reviewed by (Johnson and Gilbert, 2015). In the current study, the same host/AMF genotype combination was used as in the previous study (Cabral et al., 2018), only the biotic stress-inducer changed from a pest to a fungal pathogen. Thus, the efficiency of the signalling through the AMF hyphosphere, as well as the changes in primary metabolic activities might be dependent of host/AMF/stressor combinations, which could be related to the changes observed in the hyphosphere microbiota composition and their activities as PGPRs and biocontrol agents (Welc et al., 2010; Battini et al., 2016).

In the infected plants, the relative expression of *PR1* was higher in plants infected with *B. fabae* at an earlier time point than in previous studies (Babikova et al., 2013; Cabral et al., 2018), remaining consistently higher in infected plants, when compared to the non-infected. *PR1* is a well known SA-dependent marker (Gruner et al., 2013), which could explain the higher and faster relative expression in plants infected with a fungal pathogen, when compared to aphid infestation (Babikova et al., 2013; Cabral et al., 2018). The results presented in this study are also in agreement with the ones presented by Song et al. (2010), where the increase in relative expression of *PR1* follows the same pattern, however, peaking at a later time point than in the current study, which might be due to the different combination of AMF genotype, plant species and fungal pathogen. In the interconnected neighbouring plants, the relative expression of *PR1* was higher in interconnected plants at 48 hpi, peaking 60 at hpi, which was relatively faster than in the previous studies (Babikova et al., 2013; Cabral et al., 2018). However, the time that elapsed between the increase in *PR1* expression in infected and interconnected neighbouring plants was similar (12 h), which may indicate that the earlier induction of defence response in interconnected neighbouring plants was likely due to the higher and faster induction in infected plants.

*PR2* had higher relative expression in plants infected with *B. fabae* at 48 hpi and all later time points, which can be related to its glucanase activity and its reported induction in faba bean after *B.fabae* infection (El-Komy, 2014). *PR5* was also upregulated in infected plants at 48 hpi, following the same pattern as the other PR protein-coding genes, which is consistent with the PR5 protein functions inside the plant (Gruner et al., 2013).In interconnected neighbouring plants, the relative expression of *PR2* was higher already at 24 hpi and all subsequent time points, while the relative expression of *PR5* also peaked in interconnected neighbouring plants as soon as 24 hpi, remaining higher at 48 hpi and decreasing thereafter. The early increases in *PR2* and *PR5* relative expressions in neighbouring plants were unexpected, as no significant increase seemed to occur at 24 hpi in infected plants. As *PR2* and *PR5* have been classified as partly-dependent-SA genes (Gruner et al., 2013), in contrast to the SA-dependent *PR1*, this increase in relative expression in interconnected neighbouring plants might be due to some other type of signalling, or signalling pathway of the upregulation in the expression of these PR proteins in neighbouring plants. Moreover, due to its glucanase activity, the PR2 protein has also been reported to release short glucan fragments from the pathogen cell walls, which can act as signals towards further defence responses (Mauch et al., 1988; El-Komy, 2014), and this might not be entirely dependent on the SA defence pathway (Eder and Cosio, 1994). However, further hourly evaluations of expression of *PR2* and *PR5* prior to 24 hpi would help clarify, whether this upregulation of these PR proteins is due to another inducer, and not just to induction at an earlier time point in the interconnected infected plants.

The changes in hyphosphere primary metabolic profiles in the current study differed from those in our previous study, where *Aphis fabae* was used as a stressor (Cabral et al., 2018), confirming our hypothesis that changes to metabolic activity in the AMF hyphosphere depend on the type of stress applied to the challenged plants. In our previous study, an increase of hexose levels in the interconnected infested hyphosphere could be found, whereas in the current study a general decrease of the level of mannitol was detected. As the induction of the plant defence response was also relatively faster than in previous studies (Song et al., 2010; Babikova et al., 2013; Cabral et al., 2018), it might be that the metabolic changes previously observed in the hyphosphere occurred at an earlier time point, and therefore could not be detected at harvest time in this study. A time-course sampling of the hyphosphere compartments, using micro-probes would allow for both a finely-tuned observation of these changes and catalogue them from the onset of infection. Mannitol has been widely reported for its role in both osmotic and oxidative stress in plants and fungi, due to its possible role as a compatible solute and also due to its antioxidant activity (Patel and Williamson, 2016). Furthermore, its role in plant-pathogen interactions, along with that of mannitol dehydrogenase (MTD), has spiked interest, as several pathogens such as *B. cinerea* have been reported to secrete mannitol as a self-defence mechanism against (a)biotic stresses by quenching ROS in plant cells (Patel and Williamson, 2016). The activity of MTD has been shown to be repressed by sugar hexoses (Prata et al., 1997), which would allow for an accumulation of mannitol, allowing for additional use as a reserve carbohydrate. However, a de-repression of MTD in sink tissues, under high energy and carbon demand, might make mannitol readily available as a source for maintenance of the central metabolism (Williamson et al., 2002). Although mannitol has not been found in axenic cultures of the AM external mycelium (Bago et al., 2003), the reported decrease in mannitol levels in the hyphosphere in the current study might be related to the TCA cycle (Bago et al., 2002, 2003). Furthermore, mannitol is present in a wide range of plant roots, fungi and bacteria (Williamson et al., 2002; Saia et al., 2015; Patel and Williamson, 2016), which could indicate that other hyphosphere microbes contributed to the mannitol pool found in the current study. Moreover, in the current study, as the decrease in mannitol levels has been general, this might be due to an impact of the infected plant in the hyphosphere microbes, which might have been independent of AMF. Measurements of MTD activity from the onset of plant pathogen infection, combined with deeper investigations, such as sequencing of the microbiota present in the hyphosphere would be needed to elucidate the possible role of mannitol in the interconnected hyphosphere of infected plants.

Several actinobacteria strains have been reported to be associated with AMF (Lasudee et al., 2018). In the current study, actinobacteria have been found to be present in the hyphosphere both in presence and absence of AMF, indicating that the actinobacteria present were not exclusively mycorrhiza-associated (Battini et al., 2016; Lasudee et al., 2018). The decrease of signature fatty acids for actinobacteria, which are gram positive bacteria, in the AMF hyphosphere of *B. fabae* infected plants, interestingly, was not detected on the relative level of gram-positive bacteria, in contrast to the previous study (Cabral et al., 2018), where the levels of gram positive bacteria were higher in the presence of AMF. Several studies have reported both suppressive and synergistic effects on the hyphosphere microbial community due to the presence of AMF (Welc et al., 2010; Battini et al., 2016). Furthermore, a previous study reported an increase in the density of specific Gram-positive species was observed associated with the mycelium of *R. irregularis* (formerly *G. intraradices*), as is the AMF species used in the current study (Mansfeld-Giese et al., 2002). The reported differences in the hyphosphere microbial community seem to be dependent on the abiotic conditions of the specific studies (Welc et al., 2010; Cabral et al., 2018), as well as the functional compatibility between the different host genotypes/AMF species (Mansfeld-Giese et al., 2002; Albertsen et al., 2006), which might explain the differences observed in the current study. However, in contrast to the general decrease in the level of mannitol, the decrease in the levels of actinobacteria was detected only in the AM hyphosphere of *B. fabae* infected plants. This might be related to changes in secondary metabolism within the hyphosphere of infected plants, and not be directly associated to primary metabolism. Further evaluation of the secondary metabolic profiles of the hyphosphere could help shed light into these changes.

In conclusion, the present study shows that an induction of the defence response in infected plants, after *B. fabae* infection in the leaves of *Vicia faba*, resulted in a corresponding induction of the defence response in AMF-interconnected neighbouring plants. This was accompanied by changes in the primary metabolic activity of the hyphosphere and its microbiota. The induction of defence related genes in neighbouring plants occurred faster than in previous studies using other genotype combinations of plants, AMF and pest/pathogens. Also, changes to the primary metabolic profile in the hyphosphere of pathogen-infected plants were different from those in our previous study with the same plants and AMF genotypes, but challenged with aphids (Cabral et al., 2018). Finally, the observed changes at the hyphosphere microbiota level differed from the ones reported in our previous study, correlating with the different changes in primary metabolic activity.

Overall, the observed differences in biological activity between combinations of host genotype/AMF and pest/pathogen raises the question of a possible universal stress response. The perceived changes of the hyphosphere microbiota evidence the need for investigations into both the changes in primary and secondary metabolic profiles of the hyphosphere, as well as for studies of possible changes to the composition of the microbiota. Finally, fine-tuned hourly evaluations, using microprobes, would allow for a better evaluation of transient changes in gene-expression, as well as possible changes in metabolic activities. Conversely, as the nature of signalling molecules likely to travel the hyphosphere are still to be discovered, questions can also be put forth with regards to plant perception and sensing. For example, is it a signalling molecule that induces defence responses in the neighbouring plants, or is it the perception of the changes in metabolic activity in the hyphosphere that jumpstart its defence programme? Direct manipulation of the hyphosphere might be able to address these issues in order to pinpoint the nature of the signalling events traversing the hyphosphere.

## Author Contributions

CC, BW and SR designed and planned the research. CC performed the experimental work, collected data and performed data and statistical analysis. CC and CA adapted the primary metabolite extraction methods. CC wrote the manuscript with contributions from BW, CA, and SR.

## Conflict of Interest Statement

The authors declare that the research was conducted in the absence of any commercial or financial relationships that could be construed as a potential conflict of interest.

## Footnotes

1 http://gmd.mpimp-golm.mpg.de/

## References

[B1] Albertsen A. Ravnskov S. Green H. Jensen D. F. Larsen J. (2006). Interactions between the external mycelium of the mycorrhizal fungus *Glomus intraradices* and other soil microorganisms as affected by organic matter. *Soil Biol. Biochem.* 38 1008–1014. 10.1016/j.soilbio.2005.08.015

[B2] Aoun M. (2017). Host defense mechanisms during fungal pathogenesis and how these are overcome in susceptible plants: a review. *Int. J. Bot.* 13 82–102. 10.3923/ijb.2017.82.102

[B3] Azcón-Aguilar C. Barea J. (1997). Arbuscular mycorrhizas and biological control of soil-borne plant pathogens-an overview of the mechanisms involved. *Mycorrhiza* 6 457–464. 10.1007/s005720050147

[B4] Babikova Z. Gilbert L. Bruce T. J. Birkett M. Caulfield J. C. Woodcock C. (2013). Underground signals carried through common mycelial networks warn neighbouring plants of aphid attack. *Ecol. Lett.* 16 835–843. 10.1111/ele.12115 23656527

[B5] Bago B. Pfeffer P. E. Abubaker J. Jun J. Allen J. W. Brouillette J. (2003). Carbon export from arbuscular mycorrhizal roots involves the translocation of carbohydrate as well as lipid. *Plant Physiol.* 131 1496–1507. 10.1104/pp.102.007765 12644699 PMC166909

[B6] Bago B. Pfeffer P. E. Zipfel W. Lammers P. Shachar-Hill Y. (2002). Tracking metabolism and imaging transport in arbuscular mycorrhizal fungi. Metabolism and transport in AM fungi. *Plant Soil* 244 189–197. 10.1007/978-94-017-1284-2_18

[B7] Barea J.-M. Azcón R. Azcón-Aguilar C. (2002). Mycorrhizosphere interactions to improve plant fitness and soil quality. *Antonie van Leeuwenhoek* 81 343–351. 12448732 10.1023/a:1020588701325

[B8] Bati C. Santilli E. Lombardo L. (2015). Effect of arbuscular mycorrhizal fungi on growth and on micronutrient and macronutrient uptake and allocation in olive plantlets growing under high total Mn levels. *Mycorrhiza* 25 97–108. 10.1007/s00572-014-0589-0 25008210

[B9] Battini F. Cristani C. Giovannetti M. Agnolucci M. (2016). Multifunctionality and diversity of culturable bacterial communities strictly associated with spores of the plant beneficial symbiont *Rhizophagus intraradices*. *Microbiol. Res.* 183 68–79. 10.1016/j.micres.2015.11.012 26805620

[B10] Benjamini Y. Hochberg Y. (1995). Controlling the false discovery rate: a practical and powerful approach to multiple testing. *J. R. Stat. Soc. Ser. B (Methodological)* 57 289–300. 10.1111/j.2517-6161.1995.tb02031.x

[B11] Berger S. Sinha A. K. Roitsch T. (2007). Plant physiology meets phytopathology: plant primary metabolism and plant-pathogen interactions. *J. Exp. Bot.* 58 4019–4026. 10.1093/jxb/erm298 18182420

[B12] Bolton M. D. (2009). Primary metabolism and plant defense-fuel for the fire. *Mol. Plant-Microbe Interact.* 22 487–497. 10.1094/mpmi-22-5-0487 19348567

[B13] Bravo A. Brands M. Wewer V. Dörmann P. Harrison M. J. (2017). Arbuscular mycorrhiza-specific enzymes FatM and RAM2 fine-tune lipid biosynthesis to promote development of arbuscular mycorrhiza. *New Phytol.* 214 1631–1645. 10.1111/nph.14533 28380681

[B14] Brundrett M. C. Tedersoo L. (2018). Evolutionary history of mycorrhizal symbioses and global host plant diversity. *New Phytol.* 220 1108–1115. 10.1111/nph.14976 29355963

[B15] Cabral C. Ravnskov S. Tringovska I. Wollenweber B. (2016). Arbuscular mycorrhizal fungi modify nutrient allocation and composition in wheat (*Triticum aestivum* L.) subjected to heat-stress. *Plant Soil* 408 385–399.

[B16] Cabral C. Wollenweber B. António C. Rodrigues A. M. Ravnskov S. (2018). Aphid infestation in the phyllosphere affects primary metabolic profiles in the arbuscular mycorrhizal hyphosphere. *Sci. Rep.* 8:14442. 30262837 10.1038/s41598-018-32670-1 PMC6160425

[B17] Cameron D. D. Neal A. L. Van Wees S. C. M. Ton J. (2013). Mycorrhiza-induced resistance: more than the sum of its parts? *Trends Plant Sci.* 18 539–545. 10.1016/j.tplants.2013.06.004 23871659 PMC4194313

[B18] Chen X.-H. Zhao B. (2009). Arbuscular mycorrhizal fungi mediated uptake of nutrient elements by Chinese milk vetch (*Astragalus sinicus* L.) grown in lanthanum spiked soil. *Biol. Fertil. Soils* 45 675–678. 10.1007/s00374-009-0379-6

[B19] Eder J. Cosio E. G. (1994). “Elicitors of plant defense responses,” in *International Review of Cytology*, eds Jeon K. W. Jarvik J. (Cambridge, MA: Academic Press), 1–36. 10.1016/s0074-7696(08)62404-3

[B20] El-Komy M. H. (2014). Comparative analysis of defense responses in chocolate spot-resistant and -susceptible faba bean (*Vicia faba*) cultivars following infection by the necrotrophic fungus *Botrytis fabae*. *Plant Pathol. J.* 30 355–366. 10.5423/ppj.oa.06.2014.0050 25506300 PMC4262288

[B21] Frey-Klett P. Garbaye J. Tarkka M. (2007). The mycorrhiza helper bacteria revisited. *New Phytol.* 176 22–36. 10.1111/j.1469-8137.2007.02191.x 17803639

[B22] Garcia-Brugger A. Lamotte O. Vandelle E. Bourque S. Lecourieux D. Poinssot B. (2006). Early signaling events induced by elicitors of plant defenses. *Mol. Plant-Microbe Interact.* 19 711–724. 10.1094/mpmi-19-0711 16838784

[B23] Giovannetti M. Sbrana C. Avio L. Strani P. (2004). Patterns of below-ground plant interconnections established by means of arbuscular mycorrhizal networks. *New Phytol.* 164 175–181. 10.1111/j.1469-8137.2004.01145.x 33873483

[B24] Gruner K. Griebel T. Návarová H. Attaran E. Zeier J. (2013). Reprogramming of plants during systemic acquired resistance. *Front. Plant Sci.* 4:252. 10.3389/fpls.2013.00252 23874348 PMC3711057

[B25] Gutierrez N. Giménez M. J. Palomino C. Avila C. M. (2011). Assessment of candidate reference genes for expression studies in *Vicia faba* L. by real-time quantitative PCR. *Mol. Breed.* 28 13–24. 10.1007/s11032-010-9456-7

[B26] Johnson D. Gilbert L. (2015). Interplant signalling through hyphal networks. *New Phytol.* 205 1448–1453. 10.1111/nph.13115 25421970

[B27] Jones J. D. G. Dangl J. L. (2006). The plant immune system. *Nature* 444:323. 10.1038/nature05286 17108957

[B28] Josse J. Husson F. (2016). missMDA: a package for handling missing values in multivariate data analysis. *J. Stat. Softw.* 70 1–31. 10.18637/jss.v070.i01

[B29] Kassambara A. Mundt F. (2017). *Factoextra: Extract and Visualize the Results of Multivariate Data Analyses. R Package Version* 1.0.5. Available at: https://CRAN.R-project.org/package=factoextra (accessed December 12, 2018).

[B30] Kopka J. Schauer N. Krueger S. Birkemeyer C. Usadel B. Bergmüller E. (2005). GMD@CSB.DB: the golm metabolome database. *Bioinformatics* 21 1635–1638. 10.1093/bioinformatics/bti236 15613389

[B31] Kunkel B. N. Brooks D. M. (2002). Cross talk between signaling pathways in pathogen defense. *Curr. Opin. Plant Biol.* 5 325–331. 10.1016/S1369-5266(02)00275-3 12179966

[B32] Lasudee K. Tokuyama S. Lumyong S. Pathom-Aree W. (2018). Actinobacteria associated with arbuscular mycorrhizal funneliformis mosseae spores, taxonomic characterization and their beneficial traits to plants: evidence obtained from mung bean (*Vigna radiata*) and thai jasmine rice (*Oryza sativa*). *Front. Microbiol.* 9:1247. 10.3389/fmicb.2018.01247 29942292 PMC6004784

[B33] Le S. Josse J. Husson F. (2008). FactoMineR: an R package for multivariate analysis. *J. Stat. Softw.* 25 1–18.

[B34] Li B. Ravnskov S. Xie G. Larsen J. (2007). Biocontrol of *Pythium* damping-off in cucumber by arbuscular mycorrhiza-associated bacteria from the genus *Paenibacillus*. *BioControl* 52 863–875. 10.1007/s10526-007-9076-2

[B35] Lisec J. Schauer N. Kopka J. Willmitzer L. Fernie A. R. (2006). Gas chromatography mass spectrometry-based metabolite profiling in plants. *Nat. Protoc.* 1:387. 10.1038/nprot.2006.59 17406261

[B36] Livak K. J. Schmittgen T. D. (2001). Analysis of relative gene expression data using real-time quantitative PCR and the 2^−ΔΔCT^ method. *Methods* 25 402–408. 10.1006/meth.2001.1262 11846609

[B37] Luedemann A. Strassburg K. Erban A. Kopka J. (2008). TagFinder for the quantitative analysis of gas chromatography-mass spectrometry (GC-MS)-based metabolite profiling experiments. *Bioinformatics* 24 732–737. 10.1093/bioinformatics/btn023 18204057

[B38] Mansfeld-Giese K. Larsen J. Bødker L. (2002). Bacterial populations associated with mycelium of the arbuscular mycorrhizal fungus *Glomus intraradices*. *FEMS Microbiol. Ecol.* 41 133–140. 10.1016/s0168-6496(02)00265-9 19709247

[B39] Mauch F. Mauch-Mani B. Boller T. (1988). Antifungal hydrolases in pea tissue: II. Inhibition of fungal growth by combinations of chitinase and beta-1,3-glucanase. *Plant Physiol.* 88 936–942. 10.1104/pp.88.3.936 16666407 PMC1055685

[B40] McGonigle T. P. Miller M. H. Evans D. G. Fairchild G. L. Swan J. A. (1990). A new method which gives an objective measure of colonization of roots by vesicular-arbuscular mycorrhizal fungi. *New Phytol.* 115 495–501. 10.1111/j.1469-8137.1990.tb00476.x 33874272

[B41] Meier U. Bleiholder H. Buhr L. Feller C. Hack H. Heß M. (2009). The BBCH system to coding the phenological growth stages of plants-history and publications. *J. Kulturpflanzen* 61 41–52.

[B42] Mikkelsen B. L. Rosendahl S. Jakobsen I. (2008). Underground resource allocation between individual networks of mycorrhizal fungi. *New Phytol.* 180 890–898. 10.1111/j.1469-8137.2008.02623.x 18801003

[B43] Patel T. K. Williamson J. D. (2016). Mannitol in plants, fungi, and plant-fungal interactions. *Trends Plant Sci.* 21 486–497. 10.1016/j.tplants.2016.01.006 26850794

[B44] Prata R. T. Williamson J. D. Conkling M. A. Pharr D. M. (1997). Sugar repression of mannitol dehydrogenase activity in celery cells. *Plant Physiol.* 114 307–314. 10.1104/pp.114.1.307 12223706 PMC158306

[B45] R Development Core Team (2017). *R: A Language and Environment for Statistical Computing.* Vienna: R Foundation for Statistical Computing.

[B46] Ravnskov S. Larsen J. (2016). Functional compatibility in cucumber mycorrhizas in terms of plant growth performance and foliar nutrient composition. *Plant Biol.* 18 816–823. 10.1111/plb.12465 27094118

[B47] Ravnskov S. Wu Y. Graham J. H. (2003). Arbuscular mycorrhizal fungi differentially affect expression of genes coding for sucrose synthases in maize roots. *New Phytol.* 157 539–545. 10.1046/j.1469-8137.2003.00692.x 33873403

[B48] Rozen S. Skaletsky H. (2000). “Primer3 on the WWW for general users and for biologist programmers,” in *Bioinformatics Methods and Protocols*, eds Misener S. Krawetz S. A. (Berlin: Springer), 365–386. 10.1385/1-59259-192-2:365 10547847

[B49] Saia S. Ruisi P. Fileccia V. Di Miceli G. Amato G. Martinelli F. (2015). Metabolomics suggests that soil inoculation with arbuscular mycorrhizal fungi decreased free amino acid content in roots of durum wheat grown under N-limited, P-rich field conditions. *PLoS One* 10:e0129591. 10.1371/journal.pone.0129591 26067663 PMC4466249

[B50] Schauer N. Steinhauser D. Strelkov S. Schomburg D. Allison G. Moritz T. (2005). GC–MS libraries for the rapid identification of metabolites in complex biological samples. *FEBS Lett.* 579 1332–1337. 10.1016/j.febslet.2005.01.029 15733837

[B51] Smith S. E. Smith F. A. (2011). Roles of arbuscular mycorrhizas in plant nutrition and growth: new paradigms from cellular to ecosystem scales. *Annu. Rev. Plant Biol.* 62 227–250. 10.1146/annurev-arplant-042110-103846 21391813

[B52] Song Y. Y. Ye M. Li C. He X. Zhu-Salzman K. Wang R. L. (2014). Hijacking common mycorrhizal networks for herbivore-induced defence signal transfer between tomato plants. *Sci. Rep.* 4:3915. 24468912 10.1038/srep03915 PMC3904153

[B53] Song Y. Y. Zeng R. S. Xu J. F. Li J. Shen X. Yihdego W. G. (2010). Interplant communication of tomato plants through underground common mycorrhizal networks. *PLoS One* 5:e13324. 10.1371/journal.pone.0013324 20967206 PMC2954164

[B54] Staats M. Van Baarlen P. Van Kan J. A. L. (2005). Molecular phylogeny of the plant pathogenic genus botrytis and the evolution of host specificity. *Mol. Biol. Evol.* 22 333–346. 10.1093/molbev/msi020 15496556

[B55] Vannette R. L. Hunter M. D. (2009). Mycorrhizal fungi as mediators of defence against insect pests in agricultural systems. *Agric. For. Entomol.* 11 351–358. 10.1111/j.1461-9563.2009.00445.x

[B56] Vierheilig H. Coughlan A. P. Wyss U. Piché Y. (1998). Ink and vinegar, a simple staining technique for arbuscular-mycorrhizal fungi. *Appl. Environ. Microbiol.* 64 5004–5007. 9835596 10.1128/aem.64.12.5004-5007.1998 PMC90956

[B57] Wei T. Simko V. (2017). *R Package “Corrplot”: Visualization of a Correlation Matrix (Version 0.84*) (accessed December 12, 2018).

[B58] Welc M. Ravnskov S. Kieliszewska-Rokicka B. Larsen J. (2010). Suppression of other soil microorganisms by mycelium of arbuscular mycorrhizal fungi in root-free soil. *Soil Biol. Biochem.* 42 1534–1540. 10.1016/j.soilbio.2010.05.024

[B59] Weremijewicz J. Sternberg L. S. Janos D. P. (2016). Common mycorrhizal networks amplify competition by preferential mineral nutrient allocation to large host plants. *New Phytol.* 212 461–471. 10.1111/nph.14041 27265515

[B60] Whipps J. M. (2004). Prospects and limitations for mycorrhizas in biocontrol of root pathogens. *Can. J. Bot.* 82 1198–1227. 10.1139/b04-082

[B61] Wickham H. (2010). *ggplot2: Elegant Graphics for Data Analysis (Use R!).* New York, NY: Springer.

[B62] Williamson J. D. Jennings D. B. Guo W.-W. Pharr D. M. Ehrenshaft M. (2002). Sugar alcohols, salt stress, and fungal resistance: polyols-multifunctional plant protection? *J. Am. Soc. Hortic. Sci.* 127 467–473. 10.21273/jashs.127.4.467

