# Supplementary material for: Activity in the Arbuscular Mycorrhizal Hyphosphere Warning Neighbouring Plants
Source: Front Plant Sci. 2019 Apr 18;10:511. doi: 10.3389/fpls.2019.00511 (PMC6482268; doi:10.3389/fpls.2019.00511)
Supplement: Supplementary file 1 [file Table_1.DOCX]

Supplementary Material

**Supplementary Table 1 -** Growth parameters of *Vicia faba* L. plants after harvest.

|  | **Challenged plants** | | **Neighbouring plants** | |  |
| --- | --- | --- | --- | --- | --- |
| **Growth parameters** | **M-** | **M+** | **M-** | **M+** | **AMF** |
| **Shoot Length (m)** | 0.98±0.04a^1^ | 0.86±0.04a | 0.88±0.03a | 0.90±0.02a | ns |
| **Shoot dry weight (g)** | 10.07±0.52a | 9.71±0.49a | 9.38±0.34a | 9.96±0.44a | ns |
| **Root dry weight (g)** | 0.95±0.05a | 0.912±0.02a | 0.95±0.4a | 1.12±0.07a | ns |

Challenged plant columns represent plants from the central compartment inoculated (M+) or not (M-) with arbuscular mycorrhizal fungi (AMF). where both plants infected with *Botrytis fabae* (B+) or not (B-) are grouped. Neighbouring plant columns represent plants from the neighbouring compartments. in which AM networks via the donor plant were (M+) or not (M-) established.

Values are means ± standard error (SE) (Challenged plants: N=10; Neighbouring plants: N=10). Statistical significance between treatments indicated by * (*P*< 0.05). ** (*P<* 0.01). *** (*P*< 0.001). ns (not significant).

^1^In each row. followed by the same letter are not significantly different by Tukey’s honestly significant difference with a *P*-value BH-fdr (Benjamini-Hochberg false discovery rate) correction.

**Supplementary Figure 1 -** Standard curve for the *Botrytis fabae* quantification on leaves of inoculated (B+) challenged plants. The standard curve represents known quantities of Log10 *B.fabae* DNA and the correspondent Ct values from the qPCR reactions.


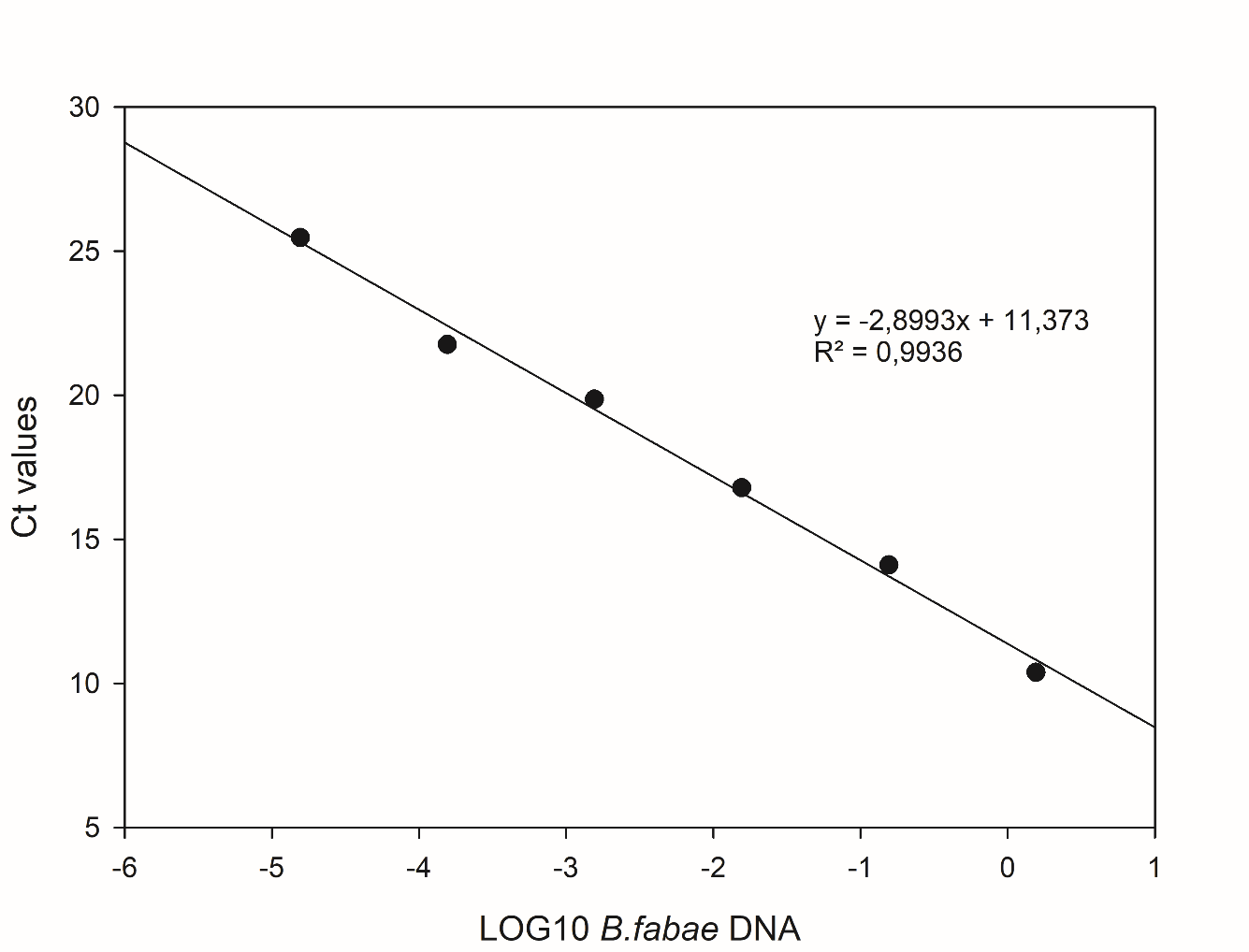


**Supplementary Table 2 -** Primer sequences used in the study.

| Gene | Primer sequence (5′–3′) | Target gene |
| --- | --- | --- |
| *VfPR1-f*  *VfPR1-r* | CAGTGGTGACATAACAGGAGCAG  CATCCAACCCGAACCGAAT | *Pathogenesis-related protein* 1  (Gutierrez *et al.*. 2011) |
| *VfPR2-f*  *VfPR2-r* | CCAATGGGTACAAAGAAACG  AAACCAAGTAACCAATGAAAGG | *Pathogenesis-related protein 2*  (El-Komy. 2014) |
| *VfPR5-f*  *VfPR5-r* | TGCCTTAGCTTTGCATTCCT  TGAGGCTTTTGGTCTCCAGT | *Pathogenesis-related protein* 5  Genbank. JQ043350.1 |
| *VfELF1A-f*  *VfELF1A-r* | GTGAAGCCCGGTATGCTTGT  CTTGAGATCCTTGACTGCAACATT | *Elongation factor 1-α*  (Gutierrez *et al.*. 2011) |
| *Bfabae-f*  *Bfabae-r* | GGTAGACCTCCCACCCTTGT  CGATGCCAGAACCAAGAGAT | *Botrytis fabae* 5.8 rRNA *gene* Genbank. KX074007 |
| *G3PDH-f*  *G3PDH-r* | ATTGACATCGTCGCTGTCAACGA  ACCCCACTCGTTGTCGTACCA | *Botrytis fabae G3PDH gene* Genbank. AJ705013 |
| *HSP60-f*  *HSP60-r* | CAACAATTGAGATTTGCCCACAAG  GATGGATCCAGTGGTACCGAGCAT | *Botrytis fabae HSP60 gene* Genbank. AJ716074 |

Genbank accession numbers provided when designed for use in current study. Reference publication provided when used from literature.

**Supplementary Table 3 -** Metabolomics Standards initiative Compliant Metadata

| **Metabolomics Standards Initiative Compliant Metadata** | | |
| --- | --- | --- |
| According to: | | |
| Lisec. J.. Schauer. N.. Kopka. J.. Willmitzer. L. & Fernie AR. (2006). Gas chromatography mass spectrometry-based metabolite profiling in plants. Nature Protocols. 1(1). 387–396. doi:10.1038/nprot.2006.59. | | |
| Fiehn. O. et al. (2007). Minimum reporting standards for plant biology context in metabolomics studies. Metabolomics 3(3). 195-201. doi:10.1007/s11306-007-0068-0. | | |
| Sumner. L. W. et al. (2007). Proposed minimum reporting standards for chemical analysis. Metabolomics 3(3). 211-221. doi:10.1007/s11306-007-0082-2. | | |
| "Fernie. A. R. et al. (2011). Recommendations for Reporting Metabolite Data. The Plant Cell 2(7). 2477-2482. doi ​10.​1105/​tpc.​111.​086272. | | |
| The Plant Cell July 2011 vol. 23 no. 7 2477-2482" | | |
| **BioSource** |  |  |
|  | Species | *Rhizophagus irregularis grown in 1:3 soil:sand mixture* |
|  | Genotype | not applicable |
|  | Organ | External mycelium |
|  | Organ specification | not applicable |
|  | Amount | 20g per sample |
| **Growth** |  |  |
|  | Support | 1:3 soil:sand mixture |
|  | Location | Greenhouse |
|  | Plot design | 5 randomized blocks |
|  | Light Period | 16/8 h photoperiod (500 μmol m^-2^ s^-1^ light intensity) |
|  | Humidity day | 50% |
|  | Humidity night | 70% |
|  | Temperature (day) | 22ºC |
|  | Temperature (night) | 18ºC |
|  | Watering | once daily |
|  | Nutritional | no fertilization applied to growth media |
|  | Date of plant establishment | 20-01-2017 |
| **Treatment** |  |  |
|  | Abiotic Treatment | not applicable |
|  | Biotic Treatment | *Botrytis fabae* |
|  | Dose | 3x10^5^ spores per ml |
|  | Duration | 72 hours |
| **Harvest** |  |  |
|  | Date | 15th of December 2017 |
|  | Time | 10:00 AM |
|  | Growth Stage | 9 week-old plants |
|  | Metabolism quenching | quickly frozen in liquid nitrogen (i.e. shock freezing) and stored at -80 °C |
|  | Harvest method |  |
|  | Storage | at - 80 °C until processed |
| **Sample Processing. Extraction. and Protocols** |  |  |
|  | Tissue processing | Hyphae in 1:3 soil:sand mixture was lyophilized. afterwards ground ion a ball-mill and homogenised. |
|  | Replicate sampling and analyses | 5 biological replicates |
|  | Extraction of Samples: GC-MS | methanol: water: chloroform solution containing 0.2 mg mL^-1^ ribitol (IS) was used for extracton of polar metabolites for 40 min at 70 °C. 1000 μL of the polar fraction was evaporated |
|  | Extract concentration: GC-MS | dried polar extracts were derivatized with 40 μL of 20 mg/mL of methoxyamine hydrochloride in pyridine followed by TMS derivatization using 70 μL of N-methyl-N-trimethylsilyltrifluoroacetamide and 20 μL of a mixture of fatty acid methyl esters (FAMES) |
|  | Extract clean-up | none |
|  | Extract storage | at - 80 °C |
| **GC-MS instrument** |  |  |
| *Gas Chromatography* | Sample preparation | samples were used as described above |
|  | Auto injector | MultiPurpose Sampler MPS. Gerstel. Germany |
|  | Chromatography Instrument | 6890N Agilent. Böblingen. Germany |
|  | Separation column | VF-5MS column (Varian Inc.. 30 m-lenght. 0.25 mm-inner diameter. and 0.25 μm-film thickness) |
|  | Separation parameters | the injection temperature was 230 °C. The initial temperature of the oven (85 °C) increased at a rate of 15 °C min^-1^ up to a final temperature of 360 °C |
| *Mass Spectrometry* |  |  |
|  | Instrument | Pegasus III TOF. Leco Instruments. St. Joseph. USA |
|  | Sample Introduction | GC-MS |
|  | Ionization | electron ionization |
|  | Polarity | positive ionization |
|  | Mass Analyzer | time-of-flight |
|  | Data acquisition | the transfer line to the mass spectrometer and ion source were set to 250 °C. After a solvent delay of 180 sec mass spectra were scanned from *m/z* 70-600 with the acquisition rate of 20 spectra s^-1^ |
| *Instrument performance and Method Validation* |  |  |
|  | Instrument calibration | internal mass calibration was performed using Agilent calibrant (PFTBA) |
|  | Mass Resolution | unit mass |
|  | Mass Accuracy | nominal mass |
|  | Quantification | relative quantification by internal standard and sample dry weight (using fresh weight/dry weight ratio reported in Batista-Santos et al. 2015) |
|  | Variation | based upon the internal standard |
|  | Sample Replications | 1 analytical (same analytical sample preparation) and 6 biological (same experimental condition) replicates |
|  | Internal Standard | ribitol |
|  | Quality Controls | FAMES internal standard markers and a QC control which consists of the same 1:3 soil:sand and hyphae material as each sample in every run. |
|  | Blanks | multiple blanks and wash analyses were included in the experimental design |
| *Data Preprocessing* |  |  |
|  | File format | data were exported in a net.cdf format using Agilent MSD ChemStation |
|  | Pre-processing details | data files were deconvoluted using an automated mass spectral deconvolution and identification system AMDIS (NIST. Gaithersburg. USA) |
|  | Statistics | Data was normalized by dividing each peak intensity by the corresponding internal standard peak intensity. followed by dividing by the dry weight of each sample. Log10 transformation allowed the distributions to fit the assumptions of the ANOVA (p<0.05). followed by a Tukey’s HSD comparison with an fdr p-value adjustment. All statistical analsysis were done in R software. |
| *Metabolite Identifications* |  |  |
|  | unknown compounds | not applicable |
|  | identified compounds | metabolites were identified by mass spectral matching against the published FAME MDN35 Library. from Golm Metabolome Database (http://gmd.mpimp-gol.mpg.de/download/) by using AMDIS and TagFinder 4.0 software |
